# Diffusion Imaging of Sport-related Repetitive Head Impacts—A Systematic Review

**DOI:** 10.1007/s11065-022-09566-z

**Published:** 2022-12-12

**Authors:** Inga K. Koerte, Tim L. T. Wiegand, Elena M. Bonke, Janna Kochsiek, Martha E. Shenton

**Affiliations:** 1 cBRAIN, Department of Child and Adolescent Psychiatry, Psychosomatics, and Psychotherapy, Ludwig-Maximilians-Universität, Munich, Germany; 2 Psychiatry Neuroimaging Laboratory, Department of Psychiatry, Brigham and Women’s Hospital, Mass General Brigham, Harvard Medical School, Boston, MA 02115 USA; 3 Graduate School of Systemic Neurosciences, Ludwig-Maximilians-Universität, Munich, Germany; 4 Department of Radiology, Brigham and Women’s Hospital, Mass General Brigham, Harvard Medical School, Boston, MA USA

**Keywords:** Diffusion magnetic resonance imaging, Repetitive head impacts, Contact sport, Concussion, Chronic traumatic encephalopathy, Youth athletes, Soccer, Header

## Abstract

Repetitive head impacts (RHI) are commonly observed in athletes participating in contact sports such as American football, ice hockey, and soccer. RHI usually do not result in acute symptoms and are therefore often referred to as subclinical or “subconcussive” head impacts. Epidemiological studies report an association between exposure to RHI and an increased risk for the development of neurodegenerative diseases. Diffusion magnetic resonance imaging (dMRI) has emerged as particularly promising for the detection of subtle alterations in brain microstructure following exposure to sport-related RHI. The purpose of this study was to perform a systematic review of studies investigating the effects of exposure to RHI on brain microstructure using dMRI. We used the Preferred Reporting Items for Systematic Reviews and Meta-Analyses (PRISMA) to determine studies that met inclusion and exclusion criteria across three databases. Seventeen studies were identified and critically evaluated. Results from these studies suggest an association between white matter alterations and RHI exposure in youth and young adult athletes. The most consistent finding across studies was lower or decreased fractional anisotropy (FA), a measure of the directionality of the diffusion of water molecules, associated with greater exposure to sport-related RHI. Whether decreased FA is associated with functional outcome (e.g., cognition) in those exposed to RHI is yet to be determined. This review further identified areas of importance for future research to increase the diagnostic and prognostic value of dMRI in RHI and to improve our understanding of the effects of RHI on brain physiology and microstructure.

**Supplementary Information:**

The online version contains supplementary material available at 10.1007/s11065-022-09566-z.

## Introduction

Repetitive head impacts (RHI) are commonly observed in athletes participating in contact sports such as American football, ice hockey, and soccer. While a concussion is defined by a head impact resulting in acute symptoms that generally subside over time (McCrory et al., 2017), RHI typically do not result in acute symptoms and are therefore often referred to as subclinical or “subconcussive” head impacts (Nauman & Talavage, 2018).

Over the past decade, epidemiological studies have emerged that report an association between exposure to RHI while participating in contact sports and an increased risk for the development of neurodegenerative diseases later in life (Mackay et al., 2019; McKee et al., 2013). These reports have not only garnered the interest of the media and public discussion, but, importantly, have initiated further research aimed at understanding the effects of RHI on brain structure and function. Studies have since reported alterations in neurological function as well as in cognition and behavior associated with exposure to RHI (McAllister & McCrea, 2017).

In an effort to elucidate the underlying pathophysiology of such alterations in brain function, an array of advanced neuroimaging techniques have been applied to characterize and to quantify brain alterations in those exposed to RHI (for a review see (Koerte et al., 2015)). Of note, during a hit to the head, the brain is subjected to mechanical forces which may lead to temporary shear deformation of the brain tissue (Giza & Hovda, 2001). The shear strain may result in stretching, shearing, and even tearing of axons thereby potentially disrupting brain function. Among MRI sequences applied to the study of RHI, to date diffusion-weighted MR imaging (dMRI) has emerged as particularly promising for the detection of subtle alterations in brain microstructure following exposure to sport-related RHI (Koerte et al., 2015).

Diffusion MRI is based on the quantification of diffusion properties of water molecules (Pierpaoli et al., 1996). Magnitude (diffusivity) and direction (anisotropy) of water molecule diffusion are dependent on tissue microstructure including cell size, cell density, fiber orientation, and directionality (Assaf & Pasternak, 2008; Basser & Jones, 2002; Basser & Pierpaoli, 1996; Symms et al., 2004). Commonly employed dMRI measures are fractional anisotropy (FA), mean diffusivity (MD), axial diffusivity (AD), and radial diffusivity (RD) (see Table 1). More recently, additional diffusion measures have been reported such as diffusion kurtosis imaging (DKI), and neurite dispersion and density imaging (NODDI).

**Table 1:** Definition of dMRI techniques and commonly used parameters

**Technique/Measure**	**Definition**
**Diffusion Tensor Imaging (DTI)**	Magnetic resonance imaging (MRI) technique that quantifies diffusion of water in voxels and is sensitive to damage of white matter microstructure (Basser & Pierpaoli, 1996; Koerte et al., 2015; Le Bihan et al., 2001)
** Fractional Anisotropy (FA)**	The directionality of diffusion. Values close to 0 represent isotropic diffusion (i.e., water diffusion in all directions). Values close to 1 represent anisotropic diffusion (i.e., water diffusion along a single main axis)
** Mean Diffusivity (MD)**	The average magnitude of diffusion along the three spatial axes (i.e., the amount of diffusion)
** Trace**	The summed magnitude of diffusion along the three spatial axes
** Axial Diffusivity (AD)**	The magnitude of diffusion along the main axis
** Radial Diffusivity (RD)**	The magnitude of diffusion perpendicular to the main axis (i.e., along the two orthogonal axes)
**Diffusion Kurtosis Imaging (DKI)**	While DTI considers diffusivity as a Gaussian distribution, DKI is an extension of DTI that quantifies the non-Gaussian distribution of water diffusion (i.e., the kurtosis) (Arab et al., 2018; Jensen et al., 2005)
** Mean Kurtosis (MK)**	The magnitude of diffusion kurtosis along the three spatial axes (i.e., the amount of diffusion kurtosis in a volume)
** Axial Kurtosis (AK)**	The magnitude of diffusion kurtosis along the main axis
** Radial Kurtosis (RK)**	The magnitude of diffusion kurtosis perpendicular to the main axis (i.e., along the two orthogonal axes)
**Neurite Orientation Dispersion and Density Imaging (NODDI)**	Diffusion MRI technique that models three tissue compartments of the brain (i.e., extracellular water, neurites, and extra-neurite tissue) (Fukutomi et al., 2019; Mitchell et al., 2019)
**Orientation Dispersion Index (ODI)**	The variability of neurite orientation. Values close to 0 represent parallelly oriented neurites. Values close to 1 represent randomly oriented neurites
**Neurite Density Index (NDI)**	The volume fraction of neurites in tissue

**Fig. 1:**
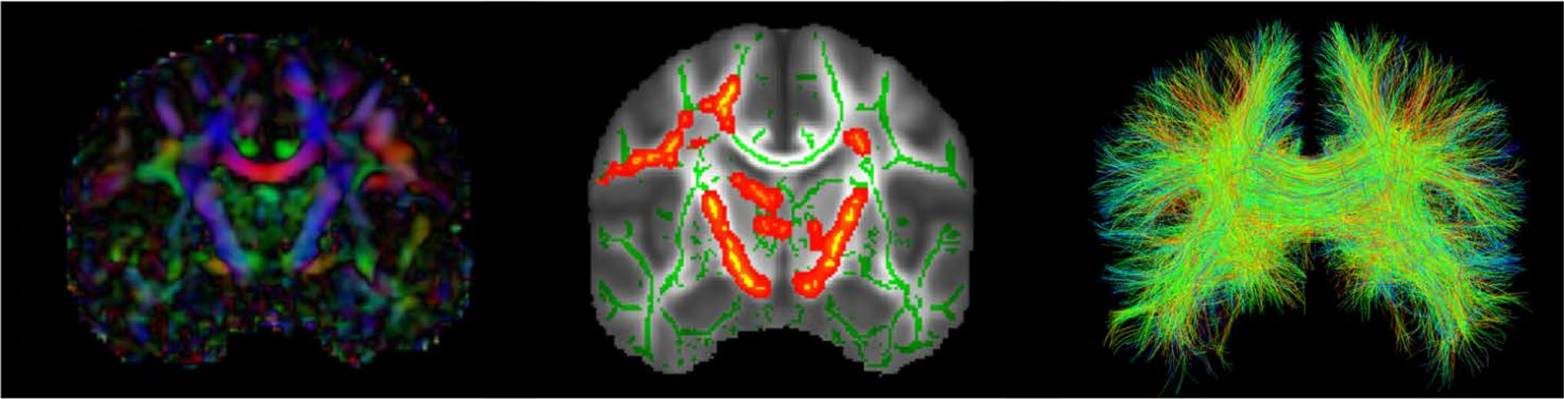
*Left*: Coronal view of a color-coded diffusion tensor map (FA) with red representing left–right, blue representing superior-inferior, and green representing anterior–posterior direction of diffusion; *Middle*: Example of a coronal view of a Tract-Based Spatial Statistics (TBSS) white matter skeleton (green) and statistically significant differences in red-yellow clusters; *Right*: Example of a coronal view of tractography of the corpus callosum using a two-tensor algorithm

Although diffusion properties of water molecules can be measured anywhere in the brain, most studies apply dMRI to the white matter using voxel-based approaches or tractography (Fig. 1). Recent histopathological studies have further underscored the link between alterations in diffusion measures and axon and myelin pathology, thereby validating the interpretation of diffusion measures as a representation of brain tissue microstructure (Budde et al., 2007; Song et al., 2003; Wiegand et al., 2021).

Here, we systematically review the literature on dMRI in individuals exposed to sport-related RHI by applying specific search terms across multiple publication data bases, and by using standardized tools for the evaluation of study quality. We summarize the literature with specific regard to the study design, sample characteristics, concussion history and the assessment of RHI, diffusion MRI sequences and analysis techniques, association with cognitive, behavioral and neurological evaluations, and associations with other biomarkers. We then draw conclusions based on the findings reported. Finally, we identify areas of importance for future research that aim to significantly increase our understanding of the effects of RHI exposure on the brain.

## Methods

### Literature Search and Study Selection

**Fig. 2:**
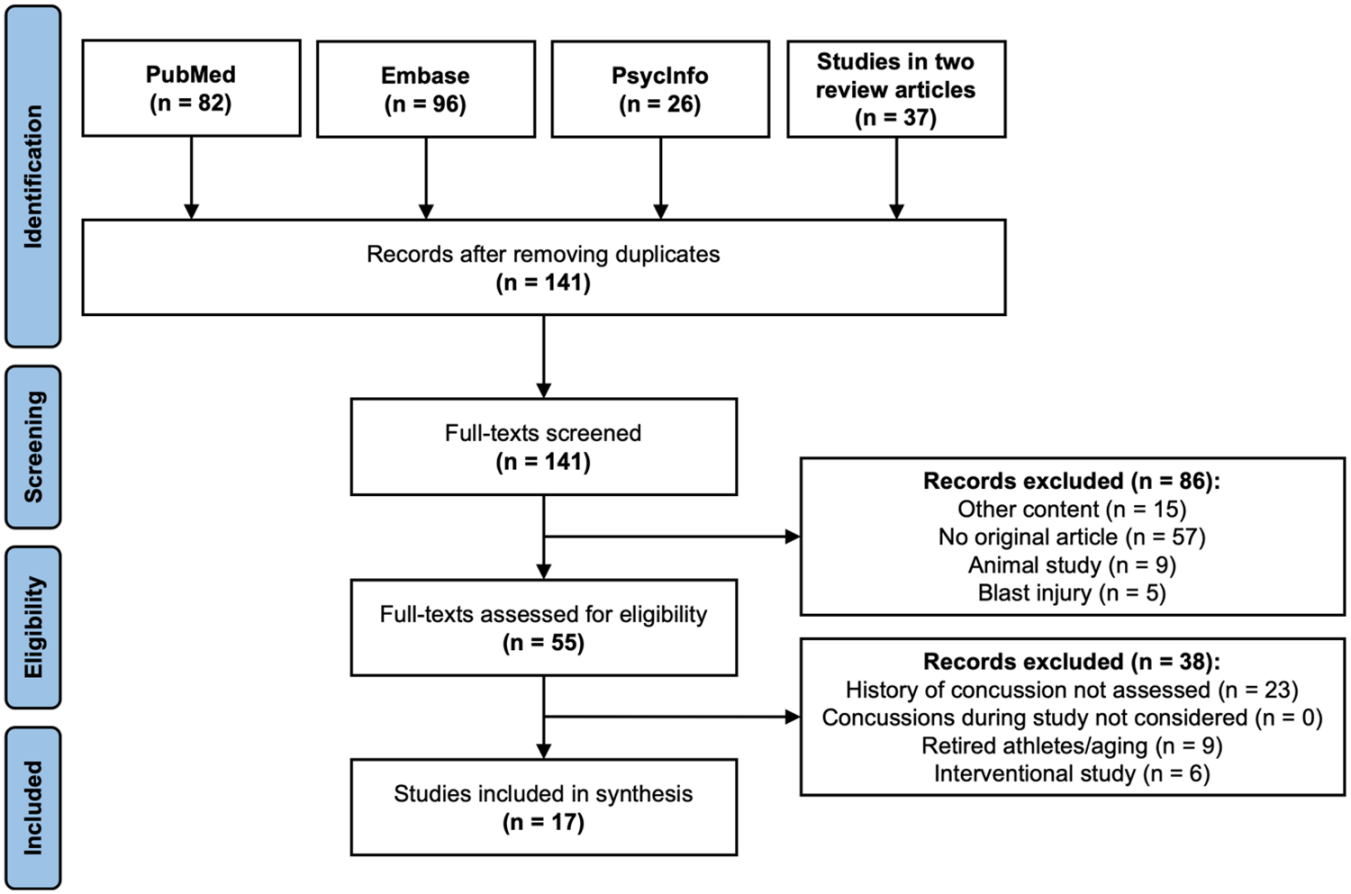
Literature search process

We followed the Preferred Reporting and Meta-Analyses (PRISMA) guidelines (Page et al., 2021) for both conducting and reporting findings for our review of dMRI findings in those exposed to RHI. Search criteria for the data base search were defined by three reviewers (IKK, TLTW, and EMB), as described in Table 2. The data base search was conducted on September 2nd, 2021 and included the search engines PubMed, Embase, and PsycINFO (see also Fig. 2). In addition, two previously published review articles (Koerte et al., 2015; Schneider et al., 2019) on neuroimaging in RHI were screened and all articles on dMRI were considered for further assessment (see Fig. 2).

**Table 2:** Search strategy for PubMed, Embase, PsycINFO and studies identified in two review articles. Numbers of articles identified on September 2^nd^, 2021 are listed for each data base

subconcuss* *OR* sub-concuss* *OR* repetitive head impact* *OR* cumulative head impact* *OR* RHI *AND* DTI *OR* diffusion tensor imaging *OR* diffusion magnetic resonance imaging *OR* diffusion imaging *OR* diffusion MRI *OR* dMRI
**PubMed**: n = 82
**Embase**: n = 96
**PsycINFO**: n = 26
**Previous Review Articles:** n = 37

Data base search and screening of previous review articles provided a total of 241 articles (see Fig. 2). After removing duplicates across the three search engines, 141 articles remained for further evaluation. From this collection, only original, peer-reviewed articles on the topic of dMRI in RHI were considered. In the first screening step, articles were excluded if the article was (a) non-original, (b) an animal study, (c) RHI was from a blast injury, or (d) reported study content other than dMRI in RHI. This process led to the exclusion of 86 articles, with 55 articles remaining. In the second step for further refinement, articles were removed if (a) concussion history was not reported, (b) concussion incidence during study participation was assessed but not adequately considered in analysis, (c) the study assessed long-term consequences in retired athletes with remote exposure to RHI, or (d) an interventional study design was used. This process led to the exclusion of 38 additional articles. The reasons for excluding each article were documented. The remaining 17 articles were included and further analyzed. Consensus on discrepancies was reached through discussion and all authors agreed on which articles should be included.

### Data Extraction and Synthesis

The following information was extracted for all 17 included articles: (a) study design, (b) sample characteristics (i.e., sample size, age, sex or gender), (c) type of contact sport, (d) type of control group, (e) concussion history and incidence during study participation, (f) dMRI sequence parameters, (g) postprocessing and analysis technique, (h) assessment of sport-related RHI, (i) findings in dMRI, (j) cognitive, behavioral, and neurological evaluations. Consensus on discrepancies was reached through discussion among the authors. Due to the heterogeneity of the methods used and the qualitative nature of the presentation of results in many of the articles, a statistical analysis or meta-analysis was not appropriate. Thus, for synthesis of findings, a narrative approach (Popay et al., 2006) was used.

### Quality Assessment

The methodological quality of studies was independently assessed by two raters (TLTW and EMB) using a QUADAS (Quality Assessment of Diagnostic Accuracy Studies) based rating which is an established tool used to systematically assess quality of diagnostic accuracy studies and to evaluate the potential risk of bias for each study (Whiting et al., 2011). QUADAS is recommended for the use in systematic reviews (Whiting et al., 2011). QUADAS-2 is the current version of QUADAS and consists of four key domains including (a) participant selection, (b) index test (i.e., dMRI), (c) reference standard (i.e., RHI), and (d) flow and timing of the study (for details see Table 3). For each of these four domains, three or five criteria were defined. An unequal number of criteria were chosen for each domain to allow for a dichotomous overall rating of each domain as either “at no risk of bias” or “at risk of bias” (for the detailed questions see Table 3). First, the raters independently rated each study based on the questions as *at no risk of bias*, *at risk of bias* or *not applicable/unclear*. Second, if more than half of the questions in one domain were answered with *at risk of bias*, this domain was rated as being *at risk of bias*. In all cases in which a question was rated as *not applicable/unclear*, the other two questions always allowed a clear total rating as *risk of bias* or *no risk of bias*. Inter-rater reliability between the two independent raters was then calculated using Cohen’s Kappa (Cohen, 1960).

**Table 3:**
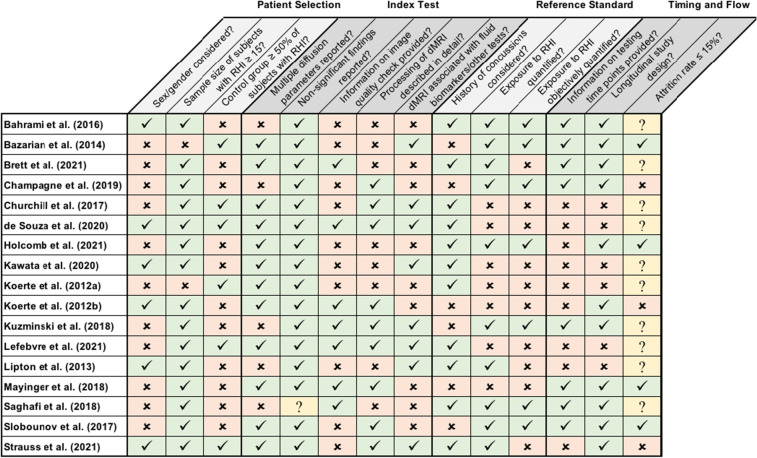
Summary of risk of bias assessment using QUADAS-2 based rating of methodological study quality

The following items were selected for reviewing the four domains. A check mark on the green background indicates that the answer to the question was “Yes”, while an X on the peach-colored background indicates that the answer to the question was “No”. A question mark on yellow background indicates that the answer was “Not applicable/Unclear”. **A) Patient Selection:** 1. Have sex differences been investigated OR were statistical analyses controlled for sex OR was sex reported in the discussion/limitations section of the manuscript? 2. Was the included number of exposed athletes sufficiently large (n ≥ 15)? 3. Has a control group been included AND was the percentage of controls ≥ 50% of those in the RHI exposed athlete sample? **B) Index Test:** 1. Were two or more dMRI measures (FA, MD, etc.) reported? 2. Have non-significant findings also been reported in the results? 3. Was a quality check of dMRI data described in detail and were measures taken appropriate for ensuring sufficient quality of data? (i.e., visual inspection of raw data, use of software for quality check of raw and processed data). 4. Was processing of dMRI data described in sufficient detail to ensure reproducibility and was software used appropriate? 5. Have diffusion measures been associated with fluid biomarkers or other tests? **C) Reference Standard:** 1. Were individuals with a history of concussion excluded OR was history of concussion considered in the statistical analyses? 2. Has exposure to RHI been quantified? 3. Has exposure to RHI been objectively quantified (e.g., counting RHI, or using sensors)? **D) Flow and Timing:** 1. Was testing time point specified and was a rationale of choice of testing time point reported? (i.e., testing time before/within/after a specific sports season and related to purpose of the study i.e., pre-postseason comparison) 2. Was attrition rate of participants ≤ 15%? 3. Was it a longitudinal study design?

## Results

**Fig. 3:**
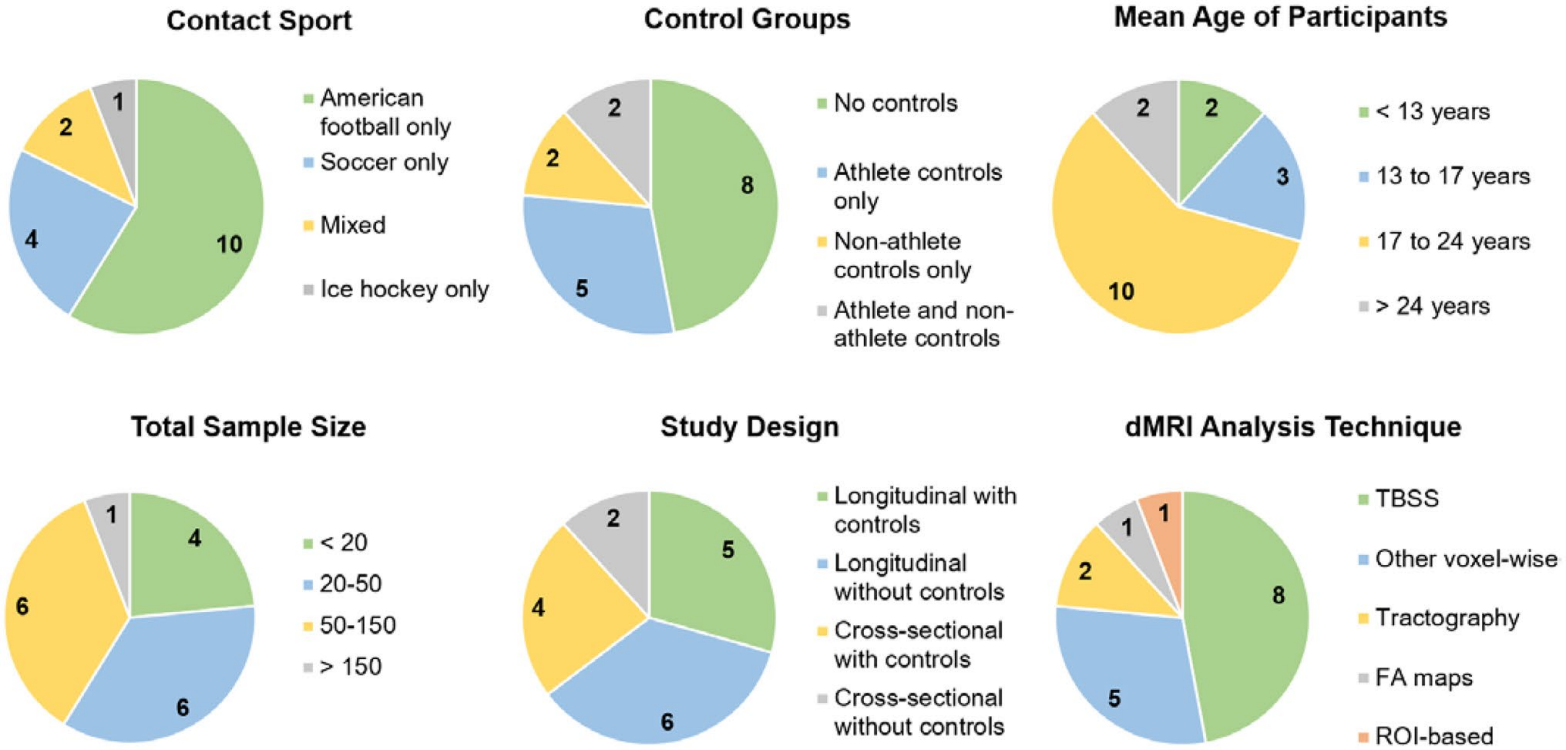
Overview of types of contact sport, types of control groups, mean age of participants, sample size, study design, and dMRI analysis technique of 17 included articles. *TBSS* Tract-Based Spatial Statistics, *FA* Fractional Anisotropy, *ROI* Region of Interest

For an overview of study characteristics, please see Fig. 3. For detailed summaries of individual studies, please see Tables 4 and 5.

**Table 4:** Summary of sample characteristics, study design, exposure to concussions and RHI, and dMRI pipeline for each included article

**Article**	**Participants** **N, (Sex, Mean Age [SD])**	**Contact Sport**	**Control Sport**	**Study Design**	**Concussions**	**Assessment of** **RHI**	**Diffusion MRI Sequence**	**Analysis** **Technique**
Bahrami et al., 2016	25 contact sport athletes (male, 11.72 [1.05] years)	American football	No controls	Longitudinal with 2 time points: Pre-season, post-season	Individuals with a history of concussion were excluded	Accelerometers	﻿﻿3 T MRI; Voxel size: 2.2 × 2.2 × 3 mm^3^; 15 diffusion directions with b = 1000 s/mm2, and 15 with b = 2000 s/mm^2^	Tractography
Bazarian et al., 2014	10 contact sport athletes (male, 20.4 [1.08] years); 5 non-athlete controls (male, 20.6 [1.14] years)	American football	Non-athlete controls	Longitudinal with 3 time points: Pre-season, post-season, 6 months of no-contact sport rest	No athlete sustained a concussion during study participation; 1 athlete had sustained a concussion > 2 weeks before study participation and was included; concussion history prior to study participation not considered in analysis	Accelerometers	3 T MRI; Voxel size: 2 × 2 × 2 mm^3^; 60 diffusion directions with b = 700 s/mm^2^	Voxel-wise analysis
Brett et al., 2021	75 American football players and 46 non-contact sport athletes (sex not specified, 18.52 [1.74] years)	American football	Non-contact sport (not further specified); were analyzed as one group together with the American football players	Longitudinal with 5 time points: baseline, after < 48 h, 8 days, 15 days, 45 days	Athletes were stratified into groups with 0, 1, and 2 + previous concussions that were analyzed	Self-report of years of contact sport exposure	3 T MRI; Voxel size: 3 × 3 × 3 mm^3^; 30 diffusion directions with b = 1000 s/mm^2^, and 30 with b = 2000 s/mm^2^	TBSS
Champagne et al., 2019	33 contact sport athletes (male, 20.3 [1.4] years)	American football	No controls	Longitudinal study with 3 time points: Pre-season, post-training camp, post-season recovery after 1 month break	Individuals with concussions during the season were excluded; athletes had sustained 0–4 concussions up to 9 years before study participation; concussion history prior to study participation not considered in analysis	Accelerometers	3 T MRI; Voxel size: 2 × 2 × 2 mm^3^; 30 diffusion directions with b = 1000 s/mm^2^	ROI-based analysis
Churchill et al., 2017	22 contact sport athletes (8 male, 14 female, 20.3 [1.5] years); 23 collision sport athletes (14 male, 9 female, 21.3 [1.9] years); 20 non-contact sport athletes (10 male, 10 female, 20.0 [1.7] years)	Contact sport: Soccer, field hockey, basketball, lacrosse, water polo; Collision sport: Rugby, ice hockey, lacrosse, American football	Volleyball	Cross-sectional at pre-season	Athletes that had sustained a concussion within 6 months prior to study participation were excluded; in athletes with a history of concussion before that, the median time since their last concussion was 2 years (range: 9 months to 8 years); athletes with previous concussions analyzed as a separate group	No quantification of RHI; exposed group	3 T MRI; Voxel size: 2 × 2 × 2 mm^3^; 30 diffusion directions with b = 700 s/mm^2^	Voxel-wise analysis
de Souza et al., 2020	17 contact sport athletes (8 male, 9 female, 19.0 [1.4] years); 39 non-contact sport athletes (20 male, 19 female, 19.3 [1.0] years)	Soccer	Non-contact sport: Tennis, basketball, volleyball, running, cross country	Cross-sectional within a month of the season start	Individuals with a history of concussion were excluded	No quantification of RHI; exposed group	3 T MRI; Voxel size: 2 × 2 × 2 mm^3^; 64 diffusion directions with b = 1100 s/mm^2^	TBSS
Holcomb et al., 2021	102 contact sport athletes (male, 11.7 [1.2] years); 16 non-contact sport athletes (male, 11.0 [1.8] years)	American football	Non-contact sport: Baseball, basketball, swimming, tennis, soccer	Longitudinal with pre-season and post-season assessments of up to 3 seasons	Individuals that sustained a concussion during and within 9 months prior to study participation were excluded; 6 football players had sustained a concussion 9 to 48 months before study participation; concussion history was included as a covariate	Accelerometers	3 T MRI; Protocol 1: Voxel size: 2.2 × 2.2 × 3 mm^3^; Protocol 2: Voxel size: 2 × 2 × 2 mm^3^; Both protocols: 15 diffusion directions with b = 2000 s/mm^2^, and 15 with b = 1000 s/mm^2^	Voxel-wise analysis
Kawata et al., 2020	17 contact sport athletes (male, 16–17 years, no mean age or SD specified)	American football	No controls	Cross-sectional at pre-season	Individuals that had sustained a concussion within 12 months prior to study participation were excluded; number of concussions before that was included as a covariate	No quantification of RHI; exposed group	3 T MRI; 6 diffusion directions with b = 500 s/mm^2^, 15 with b = 1000 s/mm^2^, 15 with b = 2000s/mm^2^, and 60 with b = 3000 s/mm^2^	TBSS
Koerte et al., 2012a	12 contact sport athletes (male, 19.7 [1.6] years); 11 non-contact sport athletes (male, 21.4 [2.8] years)	Soccer	Swimming	Cross-sectional at pre-season	Individuals with a history of concussion were excluded	No quantification of RHI; exposed group	3 T MRI; 64 directions	TBSS
Koerte et al., 2012b	25 contact sport athletes (male, 22.24 [1.59] years)	Ice hockey	No controls	Longitudinal with 2 time points: Pre-season, post-season	14 athletes had sustained at least 1 concussion before study participation; 5 athletes sustained a concussion during the season and were analyzed as a separate group	No quantification of RHI; exposed group	3 T MRI; Voxel size: 2.2 × 2.2 × 2.2mm^3^; 60 diffusion directions with 700 s/mm^2^	TBSS
Kuzminski et al., 2018	17 contact sport athletes (male, 16 [0.73] years)	American football	No controls	Longitudinal with 2 time points: Pre-season, post-season	1 athlete sustained a concussion during the study and was analyzed separately; 6 athletes reported at least one concussion prior to study participation; concussion history prior to study participation not considered in analysis	Accelerometers	3 T MRI; Voxel size: 2 × 2 × 2 mm^3^; 31 diffusion directions with b = 1000 s/mm^2^	TBSS
Lefebvre et al., 2021	23 contact sport athletes (11 male, 12 female, 22.7 years, no SD specified); 24 non-contact sport athletes (12 male, 12 female, 22.7 years, no SD specified); 24 non-athlete controls (12 male, 12 female, 22.7 years, no SD specified)	Rugby, soccer	Athlete controls (swimming); Non-athlete controls	Cross-sectional during off-season	Individuals with a history of concussion were excluded	No quantification of RHI; exposed group	3 T MRI; Voxel size: 2 × 2 × 2 mm^3^; 64 diffusion directions with b = 1000 s/mm^2^	Tractography
Lipton et al., 2013	37 contact sport athletes (28 males, 9 females, 30.9 years, no SD specified, range 21–44 years)	Soccer	No controls	Cross-sectional around off season	Lifetime number of concussions was included as a covariate	Self-report questionnaire on prior heading exposure	3 T MRI; Voxel size: 2 × 2 × 2 mm^3^; 32 diffusion directions with b = 800 s/mm^2^	Voxel-wise analysis
Mayinger et al., 2018	15 contact sport athletes (male, 20.0 [1.0] years); 5 non-athlete controls (male, 20.93 [1.1] years)	American football	Non-athlete controls	Longitudinal with 3 time points: Pre-season, post-season, 6 months of no-contact sport rest	No athlete sustained a concussion during study participation; 3 athletes had sustained a concussion > 1 year prior to study participation; concussion history not considered in statistical analysis	No quantification of RHI; exposed group	3 T MRI; Voxel size: 2 × 2 × 2 mm^3^; 60 diffusion directions with b = 1200 s/mm^2^	TBSS
Saghafi et al., 2018	122 contact sport athletes (sex not specified, 9–18 years)	American football	No controls	Longitudinal with 2 time points: Pre-season, post-season	Individuals with a history of concussion were excluded	Accelerometers	3 T MRI; Voxel size: 2.2 × 2.2 × 2.2 mm^3^; 15 diffusion directions with b = 1000 s/mm^2^	FA maps, deep learning
Slobounov et al., 2017	18 contact sport athletes (male, 21.6 [1.28] years)	American football	No controls	Longitudinal with 2 time points: Pre-season, post-season	No athlete sustained a concussion during or within 9 months prior study participation; 6 athletes had sustained one previous concussion, 2 had two previous concussions; concussion history not considered in statistical analysis	Accelerometers	3 T MRI; Voxel size: 2 × 2 × 2 mm^3^; 30 diffusion directions with b = 1000 s/mm^2^, and 30 with b = 2000s/mm^2^	TBSS
Strauss et al., 2021	246 contact sport athletes (174 male, 72 female, 25.48 [7.2] years); 72 non-collision sport athletes (30 male, 42 female, 22.76 [5.2] years): 116 non-athlete controls (63 male, 53 female, 28.96 [11.1] years)	Soccer	Non-contact sport: Baseball, swimming, tennis, running/track, gymnastics, rowing/crew, cycling, dancing, figure skating; non-athlete controls	Longitudinal with 2 time points: Baseline, and after 2 years	Lifetime number of concussions was quantified, and previous concussion history (yes/no) included as a covariate	Self-report questionnaire on prior heading exposure	3 T MRI; Voxel size: 2 × 2 × 2 mm^3^; 32 diffusion directions with b = 800 s/mm^2^	Voxel-wise analysis

*3T* 3Tesla, *MRI* Magnetic Resonance Imaging, *RHI* Repetitive Head Impacts, *ROI* Region of Interest, *SD* Standard Deviation, *TBSS* Tract-Based Spatial Statistics

**Table 5:** Summary of findings in dMRI, cognitive and behavioral evaluations, and neurological evaluations

**Article**	**Diffusion imaging**	**Cognitive and behavioral evaluation**	**Neurological evaluation**
Bahrami et al., 2016	Significant linear association between RWE_CP_ and decreased FA of left IFOF and terminal of right SLF from pre- to post-season No statistically significant association found between total RWE_CP_ and FA changes in forceps minor and major of the corpus callosum and the inferior longitudinal fasciculus	No results reported	No results reported
Bazarian et al., 2014	Significant positive correlation between percentage of voxels with FA decreasing from pre- to post-season and several helmet impact measures derived from HITS; significantly greater changes in FA and MD between pre- and post-season, and between pre- and six months post-season in contact sport athletes compared to controls; association between persistence of DTI changes between pre- and six months post-season and changes in serum ApoA1 and S100B antibodies	ImPACT; no correlations between changes in DTI between pre- and post-season; significant correlations between changes in DTI between pre- and six months post-season with both improved and worsened cognitive performance	BESS, Wii Balance Board; no correlations of balance and changes in DTI
Brett et al., 2021	Significant association between years of contact sport exposure and lower RD as well as higher FA and RK in several brain regions	No results reported	No results reported
Champagne et al., 2019	Significant decrease in FA between pre-season and post-training camp as well as pre- and one month post-season in the CC (genu and splenium) in athletes from the high exposure group; no statistically significant differences in the midsection of the CC	No results reported	No results reported
Churchill et al., 2017	Significantly higher FA and lower MD in several WM clusters in collision sport athletes, compared to contact and non-contact sport athletes at pre-season	SCAT-3; no group differences	SCAT-3; no group differences
de Souza et al., 2020	Significant association between neck strength and higher FA as well as lower RD in several WM regions at pre-season in soccer players; no significant association between MD or AD and neck strength	No results reported	No results reported
Holcomb et al., 2021	Significant positive linear relationship between percent change in FA from pre- to post-season and CMPS1 × SR in several brain regions; significant group differences in percent change in FA from pre- to post-season between high-strain football athletes and controls but not low-strain football athletes and controls	No results reported	No results reported
Kawata et al., 2020	Significant correlation between serum tau levels and elevated MD as well as lower NDI in several WM tracts; significant negative association between NfL and ODI in longitudinal fasciculus; no association between Nfl and DTI measures	No results reported	No results reported
Koerte et al., 2012a	Significantly higher RD in several WM regions, and higher AD in the CC in soccer players compared to control athletes at pre-season; no statistically significant group differences for FA and MD	No results reported	No results reported
Koerte et al., 2012b	Significant increase in trace, RD, and AD from pre- to post-season in several brain regions in contact sport athletes; no significant differences between pre- and post-season for FA	No results reported	No results reported
Kuzminski et al., 2018	Significant association between decrease of FA in the fornix-stria terminalis and cingulum-hippocampus regions (ROI analysis) from pre- to post-season and impact frequency as examined by HITS	CNS Vital Sign Assessment; neuropsychological assessment of aggression, anger and hostility, the Buss-Perry Aggression Questionnaire, anxiety, the State-Trait Anxiety Inventory, impulsivity, and the Barratt Impulsiveness Scale; significant correlation of decrease of FA in the fornix-stria terminalis region and decline in visual memory from pre- to post-season	No results reported
Lefebvre et al., 2021	Significantly higher FA in non-contact sport athletes compared to contact sport athletes in the CC and the corticospinal tract during off-season; significantly higher FA in non-contact sport athletes compared to non-athlete controls in the anterior regions of the CC and the corticospinal tracts; no group differences between contact sport and non-athlete control groups	D2 Test of Attention, Color Trails A and B, Symbol Digit Modality Test, D-KEFS' Color Word Interference Test, Rey Complex Figure Test; no group differences in cognitive performance; no correlations of cognitive performance and DTI	No results reported
Lipton et al., 2013	Significant association between number of RHI and lower FA during off-season in the temporo-occipital WM with a threshold that varied according to location (885–1550 headings per year)	Cogstate; significant association between lower FA and poorer memory scores with a threshold of 1800 headings per year; other cognitive measures were not significantly associated with headings per year	No results reported
Mayinger et al., 2018	Significant increase in trace in the brainstem and left temporal lobe as well as increase of FA in the left parietal lobe between pre- and post-season; remission to the initial status after six months of rest	ImPACT; no significant group mean declines in cognitive performance at any time point	No results reported
Saghafi et al., 2018	Based on changes in FA maps between pre- and post-season, a neural network differentiated football players with high and low RWE as derived from accelerometers with an AUROC of 85.7%	No results reported	No results reported
Slobounov et al., 2017	No longitudinal changes in DTI investigating whole brain WM divided into 48 ROIs	No results reported	No results reported
Strauss et al., 2021	Significantly greater expression of low RD, and greater expression of high FA in various brain regions in athletes with no or lower number of RHI compared to non-athlete controls	Cogstate; significantly better attention, processing speed, and memory in athletes with no or lower number of headers compared to non-athlete controls; no difference in cognitive performance between soccer players with a higher number of headers and non-athlete controls; several significant associations between volume of low and high DTI measures and cognitive performance	No results reported

*AD* Axial Diffusivity, *AUROC* Area Under the Receiver Operating Curve, *BESS* Balance Error Scoring System, *CC* Corpus Callosum, *CMPS1 × SR* Cumulative Maximum Principal Strain one Times Strain Rate, *DKI* Diffusion Kurtosis Imaging, *DTI* Diffusion Tensor Imaging, *FA* Fractional Anisotropy, *HITS* Head Impact Telemetry System, *IFOF* Inferior Fronto-occipital Fasciculus, *ImPACT* Immediate Post-Concussion Assessment and Cognitive Testing, *MD* Mean Diffusivity, *NDI* Neurite Density Index, *NfL* Neurofilament Light, *ODI* Orientation Dispersion Index, *QSM* Quantitative Susceptibility Mapping, *RD* Radial Diffusivity, *RK* Radial Kurtosis, *RWE* Risk of Concussion-weighted Cumulative Exposure (cumulative exposure to RHI assessed based on computed probability for a head impact based on each accelerative event), *RWE*_*CP*_ combined probability risk-weighted cumulative exposure (RWE based on combined probability of linear and rotational accelerations), *SCAT-3* Sport Concussion Assessment Tool 3, *SLF* Superior Longitudinal Fasciculus, *WM* White Matter

### Study Quality

For an overview of the QUADAS-2 based rating, please see Table 3. Of note, each of the studies included in this review was rated by the QUADAS-2 as having at least some risk of bias. More specifically, the main issues within each of the four domains of the QUADAS-2 were as follows: (a) Patient Selection, “Consideration of sex or gender differences is missing” (11 of 17 studies) (Bazarian et al., 2014; Brett et al., 2021; Champagne et al., 2019; Churchill et al., 2017; Holcomb et al., 2021; Koerte et al., 2012a; Kuzminski et al., 2018; Lefebvre et al., 2021; Mayinger et al., 2018; Saghafi et al., 2018; Slobounov et al., 2017), or sample was too small (i.e., ≤ 15 individuals), or control group was missing (11 of 17 studies) (Bahrami et al., 2016; Brett et al., 2021; Champagne et al., 2019; Holcomb et al., 2021; Kawata et al., 2020; Koerte et al., 2012b; Kuzminski et al., 2018; Lipton et al., 2013; Mayinger et al., 2018; Saghafi et al., 2018; Slobounov et al., 2017); (b) Index Test, “No or sparse information is provided regarding the evaluation of the quality of the imaging data” (10 of 17 studies) (Bahrami et al., 2016; Bazarian et al., 2014; Champagne et al., 2019; Churchill et al., 2017; Holcomb et al., 2021; Kawata et al., 2020; Koerte et al., 2012a; Lipton et al., 2013; Slobounov et al., 2017; Strauss et al., 2021); (c) Reference Standard, “Objective quantification of RHI is missing” (10 of 17 studies) (Brett et al., 2021; Churchill et al., 2017; de Souza et al., 2020; Kawata et al., 2020; Koerte et al., 2012a, b; Lefebvre et al., 2021; Lipton et al., 2013; Mayinger et al., 2018; Strauss et al., 2021); and (d) Flow and Timing, “No description of rationale used for choice of time points of testing” (9 of 17 studies) (Churchill et al., 2017; de Souza et al., 2020; Holcomb et al., 2021; Kawata et al., 2020; Koerte et al., 2012a, b; Lefebvre et al., 2021; Lipton et al., 2013; Strauss et al., 2021).

The overall inter-rater reliability of the QUADAS-2 based assessment of the studies between the two raters was κ = 0.90. The inter-reliability was highest for reference standard (κ = 1.00) and lowest for index test (κ = 0.81), which is considered a good reliability based on Cohen (i.e., κ = 0.80 and above) (for details on inter-rater reliability see Table 6).

**Table 6:** Agreement and inter-rater reliability (Cohen’s κ) of QUADAS-2 based rating of methodological study quality between raters TLTW and EMB

	Overall	Patient selection	Index test	Reference standard	Flow and timing
Agreement	225/238	50/51	77/85	51/51	47/51
Cohen's κ	0.90	0.96	0.81	1.00	0.87

### Study Characteristics

Of the 17 studies included, 11 had a longitudinal study design (Bahrami et al., 2016; Bazarian et al., 2014; Brett et al., 2021; Champagne et al., 2019; Holcomb et al., 2021; Koerte et al., 2012b; Kuzminski et al., 2018; Mayinger et al., 2018; Saghafi et al., 2018; Slobounov et al., 2017; Strauss et al., 2021), and six had a cross sectional study design (Churchill et al., 2017; de Souza et al., 2020; Kawata et al., 2020; Koerte et al., 2012a; Lefebvre et al., 2021; Lipton et al., 2013). Most studies related their testing time points to the start and end of a competitive sport season. Of the longitudinal studies, eight studies chose time points shortly before the beginning and shortly after the end of a sport season (Bahrami et al., 2016; Bazarian et al., 2014; Holcomb et al., 2021; Koerte et al., 2012b; Kuzminski et al., 2018; Mayinger et al., 2018; Saghafi et al., 2018; Slobounov et al., 2017). Three studies also included a third time point after a post-season break without exposure (Bazarian et al., 2014; Champagne et al., 2019; Mayinger et al., 2018). Sample sizes varied between 10 and 246 (Bazarian et al., 2014; Strauss et al., 2021) for exposed individuals, and between five and 188 for controls (Bazarian et al., 2014; Strauss et al., 2021).

### Sample Characteristics

Mean age of participants in the 17 studies ranged between 11.0 and 30.9 years (Holcomb et al., 2021; Lipton et al., 2013). Two studies included individuals younger than 13 years (Bahrami et al., 2016; Holcomb et al., 2021), three studies included individuals between 13 and 17 years (Kawata et al., 2020; Kuzminski et al., 2018; Saghafi et al., 2018), 10 included individuals between 17 and 24 years (Bazarian et al., 2014; Brett et al., 2021; Champagne et al., 2019; Churchill et al., 2017; de Souza et al., 2020; Koerte et al., 2012a, b; Lefebvre et al., 2021; Mayinger et al., 2018; Slobounov et al., 2017), and two studies included individuals older than 24 years (Lipton et al., 2013; Strauss et al., 2021). The most common contact sport was American football with 11 studies included. Of note, because American football is most commonly played by male athletes, these studies investigated male individuals only (Bahrami et al., 2016; Bazarian et al., 2014; Brett et al., 2021; Champagne et al., 2019; Churchill et al., 2017; Holcomb et al., 2021; Kawata et al., 2020; Kuzminski et al., 2018; Mayinger et al., 2018; Saghafi et al., 2018; Slobounov et al., 2017). Only five studies included mixed samples with females and males (Churchill et al., 2017; de Souza et al., 2020; Lefebvre et al., 2021; Lipton et al., 2013; Strauss et al., 2021). The remaining 12 studies included males only (Bahrami et al., 2016; Bazarian et al., 2014; Champagne et al., 2019; Holcomb et al., 2021; Kawata et al., 2020; Koerte et al., 2012a, b; Kuzminski et al., 2018; Mayinger et al., 2018; Slobounov et al., 2017) or did not specify sex or gender (Brett et al., 2021; Saghafi et al., 2018).

Regarding potential sex or gender differences, one study specifically investigated sex or gender differences (de Souza et al., 2020), two studies controlled their statistical analyses for sex or gender (Lipton et al., 2013; Strauss et al., 2021), and three studies discussed sex or gender in their limitation section (Bahrami et al., 2016; Kawata et al., 2020; Koerte et al., 2012a). Nine out of 17 studies included control groups (Bazarian et al., 2014; Brett et al., 2021; Churchill et al., 2017; de Souza et al., 2020; Holcomb et al., 2021; Koerte et al., 2012a; Lefebvre et al., 2021; Mayinger et al., 2018; Strauss et al., 2021), of which five studies included non-contact sport controls (Brett et al., 2021; Churchill et al., 2017; de Souza et al., 2020; Holcomb et al., 2021; Koerte et al., 2012a), two studies included non-athlete controls (Bazarian et al., 2014; Mayinger et al., 2018), and two studies included both (Lefebvre et al., 2021; Strauss et al., 2021). The most common non-contact sport was swimming, with four studies that included swimmers in their control group (Holcomb et al., 2021; Koerte et al., 2012a; Lefebvre et al., 2021; Strauss et al., 2021). Of note, overlap between study samples exists between the studies by Mayinger et al. (2018) and by Bazarian et al. (2014).

### Concussion History

As mentioned above, studies that did not consider concussions during study participation in their analyses were excluded during the literature search process. The way in which history of concussion was assessed and reported varied across studies. More specifically, five of 17 studies did not include individuals with a history of concussion (Bahrami et al., 2016; de Souza et al., 2020; Koerte et al., 2012a; Lefebvre et al., 2021; Saghafi et al., 2018). Six studies included individuals with a history of concussion prior to study participation, but considered this in the statistical analyses only (Brett et al., 2021; Churchill et al., 2017; Holcomb et al., 2021; Kawata et al., 2020; Lipton et al., 2013; Strauss et al., 2021). The remaining six studies included individuals with a history of concussion prior to study participation, but did not consider this information in the analyses (Bazarian et al., 2014; Champagne et al., 2019; Koerte et al., 2012b; Kuzminski et al., 2018; Mayinger et al., 2018; Slobounov et al., 2017). Among the eight studies with control groups, only two studies included controls without a history of concussion or RHI exposure (Koerte et al., 2012a; Lefebvre et al., 2021).

### Exposure to RHI

Exposure to sport-related RHI was quantified in 10 of 17 studies (Bahrami et al., 2016; Bazarian et al., 2014; Brett et al., 2021; Champagne et al., 2019; Holcomb et al., 2021; Kuzminski et al., 2018; Lipton et al., 2013; Saghafi et al., 2018; Slobounov et al., 2017; Strauss et al., 2021). Among these, seven studies used accelerometers (Bahrami et al., 2016; Bazarian et al., 2014; Champagne et al., 2019; Holcomb et al., 2021; Kuzminski et al., 2018; Saghafi et al., 2018; Slobounov et al., 2017), two studies quantified the number of head impacts using self-report questionnaires (Lipton et al., 2013; Strauss et al., 2021), and one study used self-reported years of contact sport exposure (Brett et al., 2021). In all seven studies using accelerometers, exposed athletes were American football players with accelerometers mounted in their helmets. Five of these studies used the Head Impact Telemetry System from Simbex (Bahrami et al., 2016; Bazarian et al., 2014; Holcomb et al., 2021; Kuzminski et al., 2018; Saghafi et al., 2018), one study used the BodiTrak system from HeadHealth Network (Slobounov et al., 2017), and another study used the gForce Tracker by Artaflex Inc (Champagne et al., 2019).

### Diffusion MRI Sequences and Analysis Techniques

All 17 studies used 3 Tesla MRI scanners. With regard to specific dMRI sequences, 10 studies used a voxel size of 2 × 2 × 2 mm^3^ (Bazarian et al., 2014; Champagne et al., 2019; Churchill et al., 2017; de Souza et al., 2020; Kuzminski et al., 2018; Lefebvre et al., 2021; Lipton et al., 2013; Mayinger et al., 2018; Slobounov et al., 2017; Strauss et al., 2021), two studies used 2.2 × 2.2 × 2.2 mm^3^ (Koerte et al., 2012b; Saghafi et al., 2018), one study used 2.2 × 2.2 × 3 mm^3^ (Bahrami et al., 2016), one study used 3 × 3 × 3 mm^3^ (Brett et al., 2021), one study used two different protocols with different voxel sizes (Holcomb et al., 2021), and two studies did not indicate the voxel size (Kawata et al., 2020; Koerte et al., 2012a). Number of diffusion directions ranged from six to 64 (de Souza et al., 2020; Kawata et al., 2020; Koerte et al., 2012a; Lefebvre et al., 2021). Of note, all studies except for the study by Kawata et al. used at least 15 diffusion directions (Kawata et al., 2020). With regard to the analysis technique of dMRI data, eight studies used tract-based spatial statistics (TBSS) of the whole brain white matter (Brett et al., 2021; de Souza et al., 2020; Kawata et al., 2020; Koerte et al., 2012a, b; Kuzminski et al., 2018; Mayinger et al., 2018; Slobounov et al., 2017), five studies used another voxel-wise analysis approach of the whole brain (Bazarian et al., 2014; Churchill et al., 2017; Holcomb et al., 2021; Lipton et al., 2013; Strauss et al., 2021), two studies used tractography of three intrahemispheric tracts (Bahrami et al., 2016), or of the corpus callosum (CC) and corticospinal tract (Lefebvre et al., 2021 #13), one study used FA maps of the whole brain in combination with machine learning (Saghafi et al., 2018), and one study used a region of interest (ROI)-based approach of the CC (Champagne et al., 2019). Twelve studies reported FA in combination with other tensors such as MD, RD, or AD (Bazarian et al., 2014; Brett et al., 2021; Churchill et al., 2017; de Souza et al., 2020; Holcomb et al., 2021; Kawata et al., 2020; Koerte et al., 2012a, b; Lefebvre et al., 2021; Mayinger et al., 2018; Slobounov et al., 2017; Strauss et al., 2021), five studies reported only FA (Bahrami et al., 2016; Champagne et al., 2019; Kuzminski et al., 2018; Lipton et al., 2013; Saghafi et al., 2018), one study additionally reported NODDI (Kawata et al., 2020), and one study additionally reported DKI (Brett et al., 2021).

### Diffusion MRI Findings

#### Group Differences in dMRI Cross-Sectionally (3 of 17 Studies)

Lefebvre et al. (2021) found significantly lower FA in contact sport athletes compared to non-contact sport athletes in the CC and the corticospinal tract during the off-season. Moreover, they found significantly higher FA in non-contact sport athletes compared to non-athlete controls in the anterior regions of the CC and the corticospinal tracts. Similarly during pre-season, Koerte et al. (2012a) found significantly higher RD in several white matter regions, as well as higher AD (but no difference was found for FA or MD) in the CC in soccer players compared to control athletes. In contrast, Churchill et al. (2017) found significantly higher FA and lower MD in several white matter clusters in collision sport athletes (in which body-to-body collisions are allowed), compared to contact (in which body-to-body collisions are not allowed) and non-contact sport athletes at pre-season.

#### Group Differences in dMRI Longitudinally (2 of 17 Studies)

Bazarian et al. (2014) found that contact sport athletes experienced significantly greater percentage change (i.e., either increase or decrease) in white matter FA and MD between pre- and post-season, and between pre-season and six months of no contact rest compared to controls. Moreover, the percentage of voxels with decreased FA between pre-season and post-season was positively correlated with impact measures as quantified using helmet-mounted accelerometers. Further, Holcomb et al. (2021) grouped athletes in a low-strain or high-strain group based on quantification of impacts as measured using helmet-mounted accelerometers over the course of a football season and the tissue strain rates as estimated using a finite element model. The authors report significant group differences in percent change (i.e., either increase or decrease) in white matter FA from pre- to post-season between football athletes in the high-strain group and controls but not between football athletes in the low-strain group and controls.

#### Longitudinal Changes in dMRI in Exposed Athletes only (4 of 17 Studies)

Champagne et al. (2019) found a significant decrease in FA between pre-season and post-training camp as well as pre- and post-season after one month of no contact rest in the CC in athletes from the high exposure group. Koerte et al. (2012b) found a significant increase in trace, RD, and AD (but no difference in FA) from pre- to post-season in the white matter of several brain regions in contact sport athletes. Similarly, Mayinger et al. (2018) found a significant increase in trace in the brainstem and left temporal lobe, but also an increase in FA in the left parietal lobe between pre- and post-season. Further, these researchers observed a remission to the initial status after six months of rest. Lastly, Slobounov et al. (2017) detected no longitudinal changes in diffusion measures.

#### Associations between dMRI and Exposure (8 of 17 Studies)

As mentioned above, seven dMRI and exposure studies used accelerometers to quantify head impact exposure (Bahrami et al., 2016; Bazarian et al., 2014; Champagne et al., 2019; Holcomb et al., 2021; Kuzminski et al., 2018; Saghafi et al., 2018; Slobounov et al., 2017). Holcomb et al. (2021) found a significant positive linear relationship between percent change in FA from pre- to post-season and cumulative maximum principal strain one times strain rate (CMPS1 × SR), a measure of the cumulative tensile brain strain and strain rate for one season, in several brain regions. Kuzminski et al. (2018) found a significant association between decrease of FA in the fornix-stria terminalis and cingulum-hippocampus regions from pre- to post-season and impact frequency as examined by Head Impact Telemetry System (HITS). Similarly, using the HITS, Bazarian et al. (2014) found that the percentage of voxels with decreasing FA between pre- and post-season was positively correlated with several helmet impact measures. Bahrami et al. (2016) found a significant linear relationship between combined probability risk-weighted cumulative exposure (RWE_CP_) and decreased FA of left inferior fronto-occipital fasciculus and terminal of the right superior longitudinal fasciculus from pre- to post-season. Lastly, one study combined dMRI and machine learning (Saghafi et al., 2018). Based on changes in FA maps between pre- and post-season, Saghafi et al. (2018) differentiated football players with high and low risk of concussion-weighted cumulative exposure (RWE) as derived from accelerometers with an area under the receiver operating curve (AUROC) of 85.7%.

The other three studies used self-report of number of head impacts or self-report of years of RHI exposure while participating in contact sports. For example, Lipton et al. (2013) found a significant association between number of RHI and lower FA during the off-season at three ROIs in the temporo-occipital white matter with a threshold that varied according to ROI (885–1550 head impacts per year). Strauss et al. (2021) found that athletes with no or lower number of RHI showed significantly greater expression of low RD, and greater expression of high FA in various brain regions compared to non-athlete controls. Brett et al. (2021) found a significant association between years of contact sport exposure and lower RD as well as higher FA and radial kurtosis (RK) in several brain regions.

#### Associations between dMRI and other Biomarkers (2 of 17 Studies)

Bazarian et al. (2014) found that persistence of dMRI changes between pre-season and after six months without exposure to RHI was associated with changes in serum ApoA1 and S100B antibodies which are commonly investigated blood biomarkers after brain injury. Kawata et al. (2020) combined dMRI, NODDI, and blood biomarkers at pre-season. They found a significant correlation between higher serum tau levels and higher MD as well as between higher serum tau levels and lower neurite density index (NDI) in several white matter tracts. In addition, there was a significant negative association between neurofilament light and orientation dispersion index (ODI) (but not with DTI measures) in the focal area of the longitudinal fasciculus.

### Cognitive, Behavioral, and Neurological Evaluation

With regard to cognitive and behavioral evaluations, two studies used the Cogstate test battery which is a computerized cognitive testing battery (Lipton et al., 2013; Strauss et al., 2021), two studies used the Immediate Post-Concussion Assessment and Cognitive Testing (ImPACT) (Bazarian et al., 2014; Mayinger et al., 2018), one study used the Sport Concussion Assessment Tool 3 (SCAT-3) (Churchill et al., 2017), and two studies combined several other questionnaires to assess cognitive function (Kuzminski et al., 2018; Lefebvre et al., 2021). The remaining 10 studies did not perform cognitive testing.

Among the seven studies using cognitive testing, two observed associations between longitudinal change of diffusion metrics and cognitive performance. Kuzminski et al. (2018) found a significant correlation between decreased FA in the fornix-stria terminalis region and decline in visual memory score over the season. Bazarian et al. (2014) found mixed associations between changes in diffusion measures between pre-season and six months after rest-assessment and cognitive performance.

Two studies observed associations between dMRI, RHI, and cognition cross-sectionally. In the first study, Lipton et al. (2013) found a significant association between lower FA and poorer memory scores with a threshold of 1800 soccer head impacts per year. In the second study, Strauss et al. (2021) found that athletes with no or decreased number of head impacts showed significantly better attention, processing speed, verbal and working memory compared to non-athlete controls. No difference in cognitive performance was found between soccer players with a greater number of head impacts and non-athlete controls. Lastly, they found several significant associations between volume of low and high dMRI measures and cognitive performance. Three studies found neither group differences in cognitive performance nor associations with dMRI (Churchill et al., 2017; Lefebvre et al., 2021; Mayinger et al., 2018).

With regard to neurological evaluations, Bazarian et al. (2014) used the Balance Error Scoring System (BESS) and a Wii Balance Board but found no associations between balance and changes in dMRI. Churchill et al. (2017) used the SCAT-3 but found no differences between contact or collision and non-contact sport athletes. De Souza et al. (2020) found a significant association between neck strength and higher FA as well as lower RD (but no association with MD or AD) in several white matter regions at pre-season in soccer players only. The remaining 14 studies did not perform neurological evaluations.

## Discussion

The aim of this study was to provide a systematic review of the literature on dMRI to assess the effects of exposure to RHI on brain microstructure. Below, the main results across studies are summarized and conclusions are drawn based on existing findings where we identify the limitations of previous studies, and, most importantly, identify new areas for further consideration in future studies.

## Conclusions Drawn from the Current State of the Field

Overall, study findings on dMRI in individuals exposed to RHI while participating in contact-sports compared to unexposed individuals are mixed. In addition, study designs are considerably different. However, there are patterns that emerge on closer examination. That is, many of the studies found either lower FA in those exposed to RHI in comparison with a control group, or a decrease in FA over time (e.g., pre- to post-season) in those exposed to RHI. Of note, a decrease in FA was often accompanied by an increase in MD, RD, and AD.

The most consistent finding across studies was a significant association between lower or decreased FA and greater RHI exposure among exposed athletes (five of the eight studies that investigated an association between dMRI measures and exposure to RHI). This finding may indicate a dose-dependent response relationship between head impacts and alterations in white matter microstructure. While group differences between exposed and unexposed athletes may be due to a variety of pre-existing differences between individuals participating in different types of sports (i.e., differences in cardiovascular fitness, differences in sport-specific adaptations of motor areas and networks (Meier et al., 2016)), the association between exposure and white matter diffusion alterations in studies that assess RHI exposure may provide evidence of an effect of RHI on the brain. Of note, this association seems to be independent of the type of contact sport played, as this association appears in studies on American football players as well as in studies on soccer players. However, it should be pointed out that most studies (n = 11) included were based on cohorts including American football players, which may have included a bias towards this particular type of sport (Fig. 3).

Further, while dMRI is known to be highly sensitive (Koerte et al., 2015), dMRI measures are non-specific. Additionally, both decrease and increase in FA have been interpreted as sign of injury in the context of RHI. Specifically, a decrease in FA has been interpreted as a direct mechanical injury to axon and myelin sheath as well as a sign of neuroinflammatory and neurodegenerative processes that occur over time (Shenton et al., 2012). In contrast, an increase in FA has been interpreted as, for example, due to cytotoxic edema which may indicate acute tissue injury (Shenton et al., 2012).

However, increase in FA has also been interpreted as possible adaptive growth processes such as axonal budding and gliosis due to repeated injury (Churchill et al., 2017). A few studies have thus moved to reporting the percent change in diffusion measures over time (e.g., pre- to post-season) rather than simply reporting the increase or decrease in a specific diffusion measure. These studies report that RHI exposure is associated with greater change in diffusion measures over time (Bazarian et al., 2014; Holcomb et al., 2021). Importantly, the few studies that have investigated cognition in association with dMRI have reported mixed findings. Specifically, three studies have reported decreased FA as associated with lower cognitive performance (Bazarian et al., 2014; Kuzminski et al., 2018; Lipton et al., 2013), while the three other studies did not find an association between FA and cognitive function (Churchill et al., 2017; Lefebvre et al., 2021; Mayinger et al., 2018).

In summary, participation in contact-sport and RHI exposure seem to be associated with alterations in white matter microstructure as measured using dMRI, with the most consistent finding being decreased FA in association with exposure to RHI. Whether these white matter alterations are associated with lower cognitive function needs further investigation. In the following section, we summarize the most important limitations of previous research and draw conclusions about possible avenues going forward.

## Important Limitations of Previous Research

### Sample Size

Most studies included only small sample sizes. For example, 4 of 17 studies included sample sizes of less than 20 participants, and only 7 of 17 studies included more than 50 participants. This limitation based on small sample size is surprising given the large number of athletes who regularly participate in a variety of contact sports across the world. On the other hand, among prospective clinical research studies, neuroimaging studies are both time consuming and cost intensive, which likely accounts for the relatively low number of participants.

Many pressing research questions, including whether there are effects of biological sex following exposure to RHI, could be addressed using larger samples (for review of sex differences in sports concussion see Koerte et al., 2020). One way to overcome the limitation of small sample sizes is to use large-scale study approaches applied to retrospective data analysis such as those performed by the ENIGMA consortium (Thompson et al., 2020) and by the ENIGMA sports brain injury working group (Koerte et al., 2021).

### Time Course of Brain Alterations

Of further note, studies included in this review focused on brain alterations associated with exposure to RHI in either studies with cross-sectional or longitudinal designs. Thus, the microstructural alterations observed may be due to cumulative effects of RHI sustained over the course of the season of play, but also over the course of years and even decades prior to assessment. In addition, most studies did not specify the exact timing of the assessment with regard to last physical activity or training session. This means that assessments may have been hours, days, or even weeks following the last exposure to RHI, thereby making it difficult to draw conclusive interpretations regarding the causative effect of RHI exposure on dMRI findings.

Moreover, most studies did not consider aspects of brain development. Of note here, five of the 17 studies investigated participants below the age of 18. White matter maturation reaches well into the third decade of life and dMRI measures of white matter microstructure are age dependent. In addition, there are considerable spatial differences (e.g., the cingulum develops until age 30 while the inferior longitudinal fasciculus reaches its maximum development about 10 years earlier (Aubert-Broche et al., 2013)), making statistical adjustments for age across white matter tracts difficult. Further, brain development not only depends on chronological age at time of study and on duration of observation between test time points, but also on biological sex and pubertal status as well as the interaction between age and sex. Thus, based on the current state of the literature, there is still a very limited understanding of the time course of brain alterations in the context of RHI and little is known about the interaction between white matter maturation and the effects of RHI. The latter is of great importance given the fact that the vast majority of athletes regularly exposed to RHI are under the age of 30.

### Concussion History

Athletes exposed to RHI while participating in contact sports are also at high risk for concussion (McCrory et al., 2017). While five of 17 studies excluded participants with history of concussion, the remaining studies included those with concussion prior to study participation. The exclusion of athletes with a history of concussion may have introduced a selection bias, as the number of RHI has been associated with increased risk for concussion. More specifically, by excluding those with a history of concussion, these studies may have excluded athletes with the highest exposure to RHI. Studies that included athletes with a history of concussion considered this information when performing statistical analyses. However, to date, the relationship between concussion history and exposure to RHI regarding microstructural alterations is still not fully understood. That is, it is still not known whether previous concussion history increases the susceptibility of the brain to tissue strain. Churchill et al. found a significant effect of previous concussion history on higher FA in brain regions that were also affected by RHI (Churchill et al., 2017). Thus, it is challenging to interpret alterations in diffusion measures regarding causative effects as to what is due to concussion and what is due to exposure to RHI or to the combination of the two.

## Considerations for Future Investigation

### Establish Standards for dMRI Sequence and Analysis

All studies included used a 3 T MRI machine, meaning that scanner field strength is comparable. Nonetheless, there may be slight differences in diffusion encoding between manufacturers and models. Of note, multisite studies should consider establishing methods to control scanner performance across sites.

Most importantly, however, there is substantial heterogeneity in both dMRI sequence parameters and processing techniques applied to the study of RHI. In fact, each of the 17 studies included in this review used a different set of sequence parameters including a different number of gradient directions, b-values, and voxel size, making it challenging to compare dMRI findings across studies. Nonetheless, a consensus regarding dMRI sequence parameters and specific recommendations for the application of dMRI in the study of RHI would likely increase both the quality of imaging studies as well as the comparability of findings across studies.

Further, image data processing techniques were very heterogenous across studies. First, information on whether and how image data quality was evaluated was sparse in most studies. Moreover, the majority of the studies that did report quality assessment used qualitative (e.g., visual assessment of image quality) rather than quantitative data quality measures. Given the high susceptibility of dMRI sequences to motion artifact, a surprisingly small number of data sets were reported to be excluded based on image data quality assessment. This is of particular importance given that most studies then went on to use automated analysis techniques such as TBSS where regions of interest are defined based on the merged diffusion maps of all included data sets. This means that even a single low-quality scan will affect the analysis and outcome of the entire study. Of further note, none of the studies mentioned the level of training of those who performed the quality assessment. However, providing this information is standard in studies in the field of clinical neuroradiology. This lack of appropriate quality assessment in combination with the small sample sizes included in the studies is concerning regarding the interpretation of study findings. Taken together, a consensus-based recommendation on image data quality assessment and a requirement to report details on these important processing steps are needed to significantly increase the quality of imaging studies on RHI. Further, in an effort to increase scientific rigor and reproducibility, making original data available as well as the details on processing scripts and algorithms used to process and analyze data should be strongly considered in future studies.

### Encourage Multimodal Approaches

It is important to note that diffusion measures represent mathematical calculations of diffusion in a given tissue, but they do not directly correspond to neuroanatomical structures such as axons or myelin sheaths. This means that although dMRI is highly sensitive to changes in brain microstructure, diffusion measures are non-specific. Therefore, multimodal approaches that support the interpretation of findings based on dMRI are needed to further the understanding of pathophysiological processes associated with RHI. Despite the unequivocal importance, only very few studies include multiple imaging modalities. For example, Churchill et al. (2017) combined dMRI with MR spectroscopy, which provides information on brain biochemistry. They found lower N-acetyl aspartate to creatine ratio (NAA/Cr), a marker of neuronal dysfunction or loss, in contact and collision sport athletes compared to non-contact sport athletes. The same study also applied resting state functional MRI, which revealed lower functional connectivity between brain regions in those exposed to RHI. Including two additional imaging techniques supported the interpretation of higher FA and lower MD in the context of brain dysfunction due to RHI. Champagne et al. (2019) combined dMRI with yet another imaging technique, amplified MRI (aMRI), where the latter is purported to provide information on brain tissue viscoelasticity based on the measurement of sub-voxel motion of brain tissue in response to cardiac impulses. They found significant differences in tissue stiffness along white matter tracts that was associated with differences in susceptibility to tissue strain. They concluded that a higher tissue stiffness may lead to higher vulnerability to mechanical stress due to RHI, thereby elucidating a potential risk factor for brain tissue injury.

Further, by applying complementary modalities beyond imaging, the interpretation of dMRI findings in association with exposure to RHI could be significantly improved and the underlying molecular and cellular processes further elucidated. In particular, brain-derived blood biomarkers have potential to inform interpretations of diffusion measures thereby increasing our understanding of pathophysiological processes underlying alterations in these measures (Zetterberg et al., 2013). Additionally, although fluid biomarkers of brain tissue injury are available and have been used to study traumatic brain injury, there is a surprisingly small number of studies on RHI using both neuroimaging and fluid biomarkers. However, those studies that did employ both neuroimaging and fluid biomarkers significantly improved our understanding of the pathophysiology associated with RHI. For example, Bazarian et al. (2014), found that persistence of dMRI changes was associated with serum ApoA1 and S100B antibodies, suggesting that persistent microstructural alterations are indeed a sign of brain injury. Another example is the study by Kawata et al. (2020) that combined dMRI with serum tau, a brain derived blood biomarker of brain injury. Results of this study suggest that higher MD reflects axonal injury or degeneration.

### Elucidate Interaction between RHI and Physical Activity

Physical activity and particularly aerobic exertion have been shown to increase cerebral perfusion and initial evidence suggests that it may also lead to changes in diffusion measures. In fact, McAllister et al. found an increase in MD associated with aerobic exercise (McAllister et al., 2014). Moreover, two studies in this review, included non-athlete controls in addition to a commonly used control group of non-contact sport athletes. These two studies (Lefebvre et al., 2021; Strauss et al., 2021) reported a significant difference between athletes with and athletes without RHI exposure. Interestingly, they also report that non-athlete controls did not differ from exposed athletes (Strauss et al., 2021). Findings from the two studies suggest that the positive effects of physical activity on the brain may be suppressed in athletes exposed to RHI. This is in line with a study in youth soccer players that demonstrated immediate positive effects on cognition following physical activity in youth soccer players. However, the same study found a lack of cognitive improvement over the course of a play season compared to age- and gender-matched table tennis players (Koerte et al., 2017).

Strenuous physical activity may significantly influence dMRI findings. This is of particular importance when comparing groups of athletes participating in sports with different levels of aerobic exertion. Future studies therefore need to take into consideration the effects of physical activity on brain microstructure. Finally, the interaction between brain alterations due to aerobic exercise and additional tissue strain need to be elucidated.

### Limitations

There are limitations of this systematic review that need to be considered. First, we did not conduct a meta-analysis based on data from the 17 articles included. This decision was made based on the considerable differences in study designs and the heterogeneity of dMRI acquisition parameters and post-processing techniques that were used. Second, because studies on mild traumatic brain injury (mTBI) report alterations in diffusion measures years and even decades following a concussion or mTBI (Shenton et al., 2012), we decided to specify inclusion criteria regarding history of concussion. More specifically, we required studies to report history of concussion and to account for concussions that occurred during the study (e.g., subgroup analysis or inclusion as a covariate in the statistical analysis). In doing so, we may have excluded other relevant articles. However, the articles included tended to represent the more recent research in the field of RHI. Finally, although we did not specify search criteria regarding the population, all studies that met inclusion and exclusion criteria turned out to investigate RHI exposure in athletes.

### Conclusion

This systematic review identified 17 studies that used dMRI to investigate the effects of exposure to RHI on brain microstructure. Despite considerable heterogeneity in study designs as well as in technical aspects regarding acquisition and processing of dMRI data, study results suggest white matter alterations in individuals exposed to RHI while participating in contact-sports compared to unexposed individuals. Further, in those exposed to RHI, study results point toward an association between RHI exposure and white matter alterations, particularly lower or decreased FA in several brain regions. The association between decreased FA and functional outcome (e.g., cognition) in those exposed to RHI requires further investigation. Future research needs to (a) include larger sample sizes, (b) use comparable image acquisition parameters across studies, (c) investigate sex-specific differences, (d) employ multimodal imaging approaches, (e) relate imaging findings to functional outcome (e.g., cognition, behavior, neurological function), (f) determine the time course of dMRI alterations, (g) take into account aspects of brain development in youth and young adult athletes, and (h) elucidate further the interaction between physical exercise and vulnerability to RHI. This systematic review further calls for establishing standards for the acquisition and processing of dMRI data for future studies on RHI to improve scientific rigor and reproducibility and, most importantly, to allow for the comparison of findings across studies, which will significantly increase the diagnostic and prognostic value of dMRI in RHI.

## Supplementary Information

Below is the link to the electronic supplementary material.

Supplementary file1 (DOCX 70 KB)

## Data Availability

All data and material used in this systematic review is available upon request.
